# The Minimum Data Set for Rare Diseases: Systematic Review

**DOI:** 10.2196/44641

**Published:** 2023-07-27

**Authors:** Filipe Andrade Bernardi, Bibiana Mello de Oliveira, Diego Bettiol Yamada, Milena Artifon, Amanda Maria Schmidt, Victória Machado Scheibe, Domingos Alves, Têmis Maria Félix

**Affiliations:** 1 Health Intelligence Laboratory, Ribeirao Preto Medical School, University of Sao Paulo Ribeirão Preto Brazil; 2 Brazilian Rare Disease Network Porto Alegre Brazil; 3 Medical Genetics Service, Hospital de Clinicas de Porto Alegre Porto Alegre Brazil; 4 Federal University of Rio Grande do Sul Porto Alegre Brazil; 5 Faculty of Medicine, Lutheran University of Brazil Canoas Brazil

**Keywords:** health network, health care system, minimum data set, public health, rare disease

## Abstract

**Background:**

The minimum data set (MDS) is a collection of data elements to be grouped using a standard approach to allow the use of data for clinical and research purposes. Health data are typically voluminous, complex, and sometimes too ambiguous to generate indicators that can provide knowledge and information on health. This complexity extends further to the rare disease (RD) domain. MDSs are essential for health surveillance as they help provide services and generate recommended population indicators. There is a bottleneck in international literature that reveals a global problem with data collection, recording, and structuring in RD.

**Objective:**

This study aimed to identify and analyze the MDSs used for RD in health care networks worldwide and compare them with World Health Organization (WHO) guidelines.

**Methods:**

The population, concept, and context methodology proposed by the Joanna Briggs Institute was used to define the research question of this systematic review. A total of 4 databases were reviewed, and all the processes were reported using the PRISMA (Preferred Reporting Items for Systematic Reviews and Meta-Analyses) methodology. The data elements were analyzed, extracted, and organized into 10 categories according to WHO digital health guidelines. The quality assessment used the STROBE (Strengthening the Reporting of Observational Studies in Epidemiology) checklist.

**Results:**

We included 20 studies in our review, 70% (n=14) of which focused on a specific health domain and 30% (n=6) of which referred to RD in general. WHO recommends that health systems and networks use standard terminology to exchange data, information, knowledge, and intelligence in health. However, there was a lack of terminological standardization of the concepts in MDSs. Moreover, the selected studies did not follow the same standard structure for classifying the data from their MDSs. All studies presented MDSs with limitations or restrictions because they covered only a specific RD, or their scope of application was restricted to a specific context or geographic region. Data science methods and clinical experience were used to design, structure, and recommend a fundamental global MDS for RD patient records in health care networks.

**Conclusions:**

Our study highlights the difficulties in standardizing and categorizing findings from MDSs for RD because of the varying structures used in different studies. The fundamental RD MDS designed in this study comprehensively covers the data needs in the clinical and management sectors. These results can help public policy makers support other aspects of their policies. We highlight the potential of our results to help strategic decisions related to RD.

**Trial Registration:**

PROSPERO CRD42021221593; https://www.crd.york.ac.uk/prospero/display_record.php?RecordID=221593

**International Registered Report Identifier (IRRID):**

RR2-10.1016/j.procs.2021.12.034

## Introduction

### Background

The minimum data set (MDS) is a collection of data elements to be grouped in a standard approach to allow the use of data for clinical and research purposes. An MDS is designed to capture essential data elements, which can be aggregated, at an individual level, or a combination of both, depending on the specific requirements of the registry and research objectives.

Data standardization allows for the accurate comparability of collected data and, consequently, improved generalization of findings [1]. Many countries use the MDS strategy in their health systems to standardize essential and fundamental data elements to properly record patient information and support public health planning and management. In addition, MDSs facilitate data interoperability between different health services that comprise a national health network [2]. The MDS strategy makes it possible to identify relevant health indicators in health information systems (HISs) that can serve as a basis for the definition of public policies that can influence the monitoring of diseases, organize the resources available, and improve social well-being [2].

With the high volume of fragmented data in different health services, a validated, stable, and safe MDS is essential to support the use of clinical-administrative elements and information to generate national health statistics. As noted by the World Health Organization (WHO), it can help elaborate models of service performance and patient satisfaction to meet the growing health demands of HISs worldwide. Therefore, the development of HISs relevant to public health should be able not only to generate data but also understand and manage the produced information. They should also provide indicators to represent the true health context of a given population and provide subsidies to monitor the quality of treatments offered by health services [3].

However, health data are typically voluminous, complex, and sometimes ambiguous to generate indicators that can provide knowledge and information on health. Thus, raw data must be ordered, interpreted, and transformed into information [4] that must be processed and analyzed. Many digital tools are used for processing and analysis, including machine learning algorithms, artificial intelligence, neural network applications, big data, computational ontologies, and semantic web [5].

This complexity extends further to the rare disease (RD) domain. RDs are pathologies with a low prevalence in the general population, with <50 cases per 100,000 individuals [6]. Although they affect a low percentage of people individually, such diseases are numerous, and together they can affect up to 10% of the world population; thus, they significantly impact health systems [7]. For many RDs, there is no well-structured knowledge about their diagnoses and treatments, increasing the demand for MDS strategies [8].

Many countries have initiated national plans to promote care, research, and technology in RD. These plans focus on enabling health managers to improve the services provided in a contextualized way [9]. In addition, these proposals can increase the accuracy of health decisions and reduce the fragmentation of large volumes of data, creating a solid base of information pertinent to diagnoses, treatments, and processes [10].

Owing to the complexity of the areas of knowledge related to RD, initiatives have been developed to provide informational support to health networks that provide services, care, and research in RD [11]. The best known is Orphanet, a networked platform comprising researchers from European countries. It is funded by the European Commission to increase the available knowledge base of RDs to improve care processes in this domain. Orphanet promotes and provides a structured and comprehensive database of information and knowledge about the RD domain. It also offers a validated ontology with a high standard of quality consistency and manual data auditing performed by specialists [12].

In Brazil, the Brazilian Policy for Comprehensive Care for People with Rare Diseases of the Ministry of Health of Brazil established reference services in RD that offer preventive, diagnostic, and therapeutic actions for people with rare conditions [13]. There is a need for human, technological, and infrastructure resources in the Brazilian Unified Health System, which leads to management and communication problems such as difficulties in transmitting information between the services that comprise the health network, leading to different processes and costs across the public system [14].

MDS is essential for health surveillance, providing services, and generating recommended population indicators. However, the national policy in Brazil does not support the structured use of validated MDS in health services [15]. To address this issue, the Brazilian National Network of Rare Diseases (in Portuguese: *Rede Nacional de Doenças Raras* [RARAS]) was designed to create an epidemiological surveillance structure. This network encompasses all reference services for RDs enabled in Brazil and brings together university hospitals and reference services for neonatal screening from all regions of the country [16].

The RARAS project is conducting the first nationally representative survey in Brazil on the epidemiology, clinical scenarios, diagnostic and therapeutic resources, and costs related to individuals with RD of genetic and nongenetic origin. Project managers developed their own data collection protocol in the absence of a standardized global MDS reference. Although we identified this information bottleneck in Brazil, the international literature describes a global problem with data collection, recording, and structuring in RD [17].

### Objectives

This study aimed to identify and analyze the MDSs used for RD in health care networks worldwide and compare them against WHO guidelines. The secondary objectives were to verify MDS implementation and study quality, map the collection of data elements, suggest a global fundamental MDS for RD, improve diagnostic and care processes, and optimize public planning and decision-making.

## Methods

### Research Question Definition

The population, concept, and context (PCC) methodology proposed by the Joanna Briggs Institute was used to define the research question of this systematic review. The PCC strategy is recommended as an alternative to the population, intervention, comparison, and outcome model for investigations without well-defined clinical intervention [18]. A total of four research questions were defined, preceding our central question as follows: (1) Are there standardized MDSs for RD patient records in health networks worldwide? (2) What are the data elements of each MDS? (3) What can we assess of their usefulness? and (4) Can a fundamental MDS be developed for RD networks?

These questions were formulated to help answer the following central question: “What is the minimum data set used in registries for RDs in health networks?”

To improve the transparency and reproducibility of the review, we registered it in the Prospective Register of Systematic Reviews international database under the identifier CRD42021221593 [17].

### Inclusion Criteria

The inclusion criteria were research papers (eg, full texts and conference papers), national plans, policies, industry reports, position papers, and program reports. Studies published in Portuguese, English, and Spanish were included. Owing to the specificity and novelty of the proposal, we included publications from any time. Only studies that fully described MDS for RD and its practical implementation as a health strategy were included. Furthermore, only studies that answered our research question and provided clear evidence on the subject were included.

### Exclusion Criteria

Publications without the scientific rigor to answer our research question using clear and objective evidence were excluded from this systematic review. We excluded personal editorials, studies using only theoretical approaches without practical implementation (such as studies defining operational or predictive models), and social media publications. Studies published in languages other than those listed in the inclusion criteria were also excluded. In addition, studies that used an MDS for RD but did not describe all the data elements that comprised the MDS were excluded.

### Search Strategy for Selection of Studies

The concepts used to create the search strategy came from PCC methodology and were adapted using Medical Subject Headings, the vocabulary thesaurus of the National Library of Medicine used for indexing articles, and metadata for health sciences [19].

A total of 4 databases were reviewed: PubMed, Scopus, Web of Science, and *Literatura Latino-Americana e do Caribe em Ciências da Saúde* (LILACS). We also searched for documents from the WHO and government websites for additional studies. These databases were chosen because of their robustness and relevance in the clinical and health arenas. Table 1 lists the keywords and search strings entered into the search strategy for different databases. Logical connectors were used to assign the necessary precision and refine queries.

**Table 1:** Search strategies for each of the selected databases.

Database	Search strategy
PubMed	(minimum data set OR minimum data sets OR minimum data OR minimum data set OR minimum data sets OR minimum core data) AND (rare disease OR rare diseases) AND (health network OR health networks OR health service OR health services OR health administration OR public health OR health policy OR health policies)
Scopus	“minimum dataset” OR “minimum datasets” OR “minimum data” OR “minimum data set” OR “minimum data sets” OR minimum core data AND “rare disease” OR “rare diseases” AND “health network” OR “health networks” OR “health service” OR “health services” OR “health administration” OR “public health” OR “health policy” OR “health policies”
Web of science	([ALL=((minimum dataset OR minimum datasets OR minimum data OR minimum data set OR minimum data sets OR minimum core data))] AND ALL=((rare disease OR rare diseases))) AND ALL=((health network OR health networks OR health service OR health services OR health administration OR public health OR health policy OR health policies))
LILACS^a^	(Minimum data set OR minimum data sets OR minimum data OR minimum data set OR minimum data sets OR minimum core data) AND (rare disease OR rare diseases) AND (health network OR health networks OR health service OR health services OR health administration OR public health OR health policy OR health policies)

^a^LILACS: Literatura Latino-Americana e do Caribe em Ciências da Saúde.

The Google Scholar database was also used for manual searches and searching for references to theses and dissertations. These documents are part of the gray literature because they are not published in commercial media. Searching through such sources can reduce publication bias, increase the comprehensiveness and timeliness of reviews, and provide a balanced picture of available information [20]. RD clinical experts participated in the evaluation process.

### Data Collection Processes

#### Selection of Studies

After conducting searches using the terms specified for the mentioned databases, the review authors independently screened the titles and abstracts of each retrieved article for eligibility for synthesis according to the inclusion and exclusion criteria. Next, the full texts were retrieved, and the investigators independently performed another round of reviews to determine whether these full texts met the eligibility criteria. The reviewers were not blinded to the journal titles, study authors, or associated institutions.

The points of divergence between the 2 reviewers were identified, and a third independent evaluator resolved the disparities. Search results and the process used to screen for eligibility in this phase were reported using the PRISMA (Preferred Reporting Items for Systematic Reviews and Meta-Analyses) [21] methodology to present the number of studies considered in each review process step. All resulting studies were organized in a web service environment, and duplicates were removed. We also documented the reasons for the exclusion of each article. Subsequently, we extracted the data and mapped the general characteristics and contexts of the included studies.

#### Data Extraction

We initially conducted a pilot test to extract data from the selected articles. Subsequently, we aligned our data extraction method with the PRISMA checklist [22]. A series of information to be mapped from the selected articles was defined to cover all the details of the chosen methodology.

The general characteristics and context of the selected studies were extracted and organized into tables: authors, publication year, title, study design, territorial dimension, national context, study objective or objectives, sample size, data analysis method, study population, guidelines followed, the health domain in which the MDS was deployed, and the main findings of the selected studies.

After defining the articles included and extracting their general characteristics, we analyzed these documents to identify the MDSs used in health networks. We extracted all data elements that formed each of the implemented MDSs. To do this in an organized manner, we classified the informational elements into 10 categories: eligibility, identification, diagnosis, treatment, medical consultation, comorbidity, hospitalization, examination, outcome, and others. These categories were determined according to WHO digital health guidelines [23] and practical projects underway, such as RARAS [8,16].

We organized all identified MDS data elements from each included study into one of these categories. We observed that many articles described MDS data elements with different names corresponding to the same information (ie, elements that used different terms but had the same meaning). For example, the data element that represents the information about the age at which the first symptoms of the disease were identified in diagnosed patients were described in different forms in different papers, such as “Age at onset,” “Age at first symptoms in clinically diagnosed patients,” or “Age at initial symptoms.” Clinical experts in RD identified these ambiguities, and different terms with the same meaning were aggregated as unique terms (the most commonly used), synonyms were recorded, and analyses of the terms’ frequencies were adjusted.

#### Data Categorization

We initially compiled and categorized all the findings from the primary studies included in the systematic review to develop the base structure for fundamental MDS. This preliminary categorization followed WHO guidelines and recommendations for health indicators [3,23]. Subsequently, we held structured meetings with health professionals working in RD services in the public health system and specialist researchers on data standards and terminology for health and digital health. We combined and summarized the results of these meetings through synthesis methods with the findings obtained from the selected studies to generate the fundamental MDS proposed for the RD domain.

#### Risk of Bias and Quality Assessment

Several guidelines for reporting clinical research results are available on the Equator Network website [24]. None of these recommendations focus on writing the results from a data quality perspective. The Cochrane Collaboration states that the quality assessment of the published evidence must consider the reporting of each original paper. Therefore, the quality assessment of individual studies’ findings was conducted using the STROBE (Strengthening the Reporting of Observational Studies in Epidemiology) checklist [25].

The last STROBE recommendation includes 2 topics directly relevant to data quality reporting. The first refers to the importance of measuring each data element of interest and providing data sources and details of the assessment methods, describing the comparability of the assessment methods when applicable. The second section explains how to address and treat the missing data. In addition, qualitative evaluations of the evidence were independently performed by 2 researchers. If disagreements occurred, the issue was referred to a third researcher for a decision [26].

We adopted the STROBE recommendations to specifically cover the normalization of the importance of each data set found in the reported articles and their evaluation methods regardless of study design. After normalization into data set categories and study designs, we also qualitatively compared descriptions of their features and how to overcome and map the gaps reported in these findings.

In addition, we have used the PRISMA methodology to organize and formally present the overall results obtained by comparing individual reports and to provide a transparent evaluation according to the topics described by the PRISMA checklist. These checklist items are essential for the transparent reporting of a systematic review. We addressed most of these items, except topics related to meta-analysis, which did not apply to this review [22].

### Synthesis Methods

We aimed to combine the findings of similar studies into subcategories using a consensus among specialists. We did not exclude studies with inadequate quality from the data synthesis. Descriptive statistics were used to report the frequency and proportion of the outcome measures.

The subgroup analysis method was used to describe the possible reasons for the heterogeneity of the data extracted from the selected studies and to facilitate the synthesis of information via tabulation and visual arrangement so that we could later carry out the necessary analyses.

When considering the reporting method of a single instrument, proportions indicating the percentage of studies using that instrument were calculated. Next, the selected resources were analyzed and interpreted. The main contents related to the research objective were classified as RD minimum data elements. The sections were then classified into 2 general categories, management data and clinical data, which is an efficient method for categorizing health data [27].

## Results

### Description of Selected Studies

**Figure 1:**
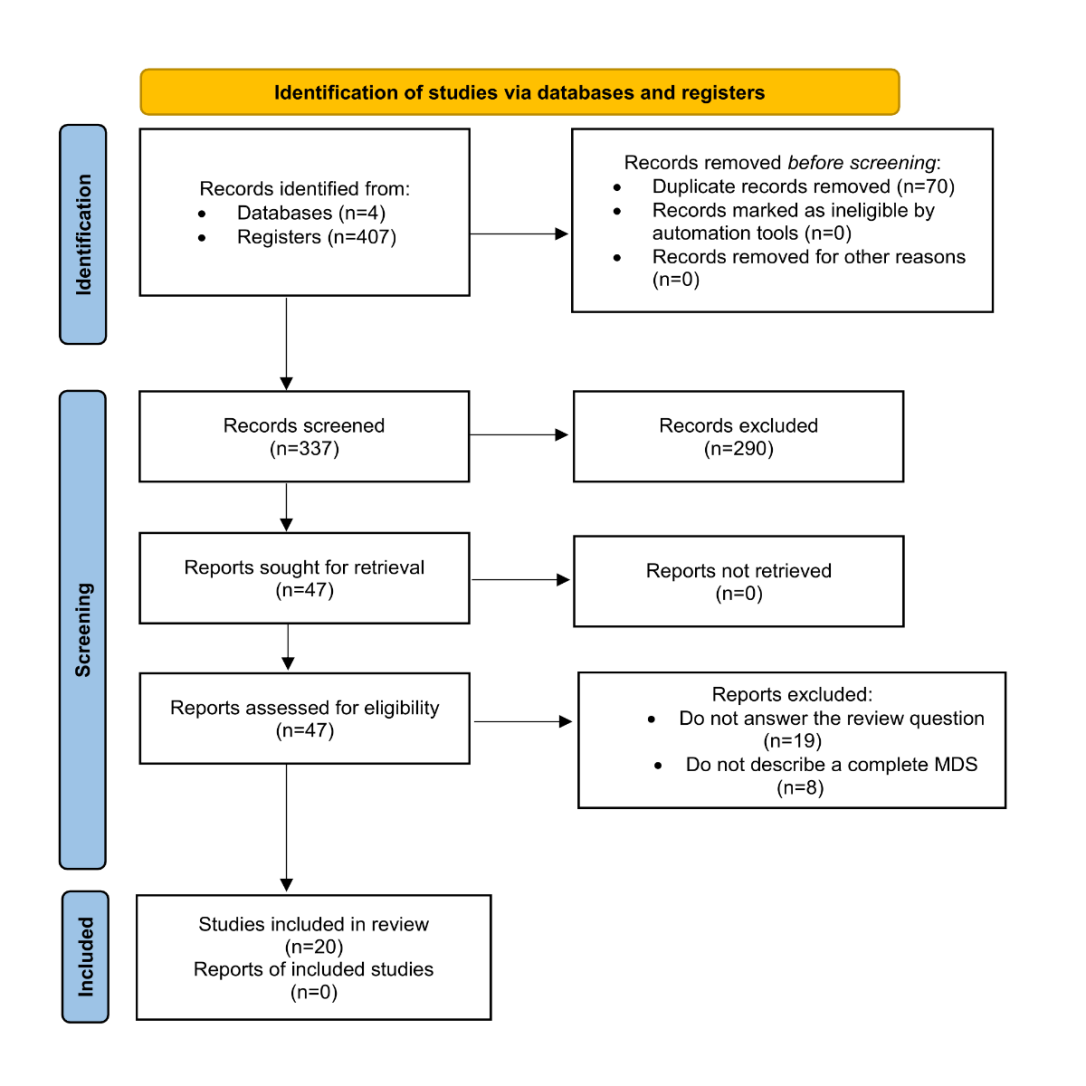
PRISMA (Preferred Reporting Items for Systematic Reviews and Meta-Analyses) flow diagram. MDS: minimum data set.

We identified 407 studies in the initial database search phase. After removing duplicates, 337 articles remained for the title and abstract screening stage, 290 of which were excluded because they were irrelevant to this review. We evaluated 47 full-text articles based on the inclusion criteria. Of these, 19 articles were excluded because they did not answer the stipulated research question, and 8 articles were excluded because they only mentioned an MDS strategy for RD and did not describe the data elements or provide information about implementation in practice. The detailed process is shown in Figure 1.

A total of 20 unique studies were included in this systematic review. The general characteristics of these studies are described in Table 2, organized by the first author’s given names in alphabetical order. The specific characteristics of these studies are described in Multimedia Appendix 1.

**Table 2:** General characteristics of selected studies.

Study, year	Study title	Study design	Territorial dimension
Berger et al [28], 2021	How to design a registry for undiagnosed patients in the framework of rare disease diagnosis: suggestions on software, data set and coding system	Applied research	Europe
Mukhina et al [29], 2020	Primary immunodeficiencies in Russia: data from the National Registry	Cohort	Russia
Licht et al [30], 2015	The global aHUS registry: methodology and initial patient characteristics	Cohort	International
Messiaen et al [31], 2008	CEMARA: a web dynamic application within a N-tier architecture for rare diseases	Case study	France
Messiaen et al [32], 2021	10 y of CEMARA database in the AnDDI-Rares network: a unique resource facilitating research and epidemiology in developmental disorders in France	Cross-sectional cohort study	France
Stanimirovic et al [33], 2019	Development of a pilot rare disease registry: A focus group study of initial steps toward the establishment of a rare disease ecosystem in Slovenia	Case study	Slovenia
Pastores et al [34], 2007	The MPS I registry: design, methodology, and early findings of a global disease registry for monitoring patients with mucopolysaccharidosis type I	Cohort	International
Stirnadel-Farrant et al [35], 2018	Gene therapy in rare diseases: the benefits and challenges of developing a patient-centric registry for Strimvelis in ADA-SCID	Case study	Italy
Tingley et al [36], 2020	Evaluation of the quality of clinical data collection for a pan-Canadian cohort of children affected by inherited metabolic diseases: lessons learned from the Canadian Inherited Metabolic Diseases Research Network	Cohort	Canada
Subirats et al [37], 2020	Biomedical holistic ontology for people with rare diseases	Applied research	Europe
Kalankesh et al [38], 2015	Minimum data set for cystic fibrosis registry: A case study in Iran	Case study	Iran
Shahmoradi et al [39], 2019	Instructional design, development and evaluation of congenital hypothyroidism registry system	Applied research	Iran
McCann et al [40], 2014	Developing a provisional, international minimal data set for Juvenile dermatomyositis: for use in clinical practice to inform research	Applied research	The United Kingdom, Italy, and Canada
McCann et al [41], 2015	Development of an internationally agreed minimal data set for JDM for clinical and research use	Applied research	International
Huemer et al [42], 2018	Phenotype, treatment practice and outcome in the cobalamin-dependent remethylation disorders and MTHFR deficiency: data from the E-HOD registry	Cohort	Europe
Choquet et al [2], 2015	A methodology for a minimum data set for rare diseases to support national centers of excellence for health care and research	Cross-sectional	France
Derayeh et al [43], 2018	National information system for rare diseases with an approach to data architecture: A systematic review	Systematic review	Europe, the United States, Australia, and Asia
Taruscio et al [44], 2015	National registries of rare diseases in Europe: an overview of the current situation and experiences	Case study	Europe
Opladen et al [45], 2021	U-IMD: the first Unified European registry for inherited metabolic diseases	Applied research	Europe
Meissner et al [46], 2021	EULAR recommendations for a core data set for pregnancy registries in rheumatology	Applied research	Europe

### Domains of Health

Of the 20 selected studies, 70% (n=14) referred to a specific health domain and the remaining 30% (n=6) referred to RD in general. Of the studies that referred to a specific domain, 2 studies were dedicated to the composition of MDS for juvenile dermatomyositis [40,41], 2 to metabolic disorders [36,45], 1 to nonmalignant RD [33], and 1 to undiagnosed RDs [28]. Other specific studies included mucopolysaccharidosis type I [34], homocystinuria and methylation defects [42], primary immunodeficiencies [29], cystic fibrosis [38], congenital hypothyroidism [39], adenosine deaminase severe combined immunodeficiency [35], atypical hemolytic uremic syndrome [30], and inflammatory rheumatic diseases [46], with 1 study each.

The remaining 6 studies that presented MDSs focused on the domain of RD in general [2,31,32,37,43,44], that is, they used data elements capable of representing patients with any RD in its health context. However, even these studies have demonstrated limitations and restrictions because of their geographic coverage and the particular local characteristics of the regions in which they were applied. As mentioned in the Discussion section, the proposed fundamental MDS seeks to address these limitations.

### Description of MDSs in the Selected Studies

All the data collected and compiled, as well as their occurrences in the MDSs of the studies included in this systematic review, have been described in Multimedia Appendix 2.

In the eligibility category, the term “patient consent” appeared the most; of the 17 terms in this category, 13 were unique. In the identification category, “patient’s name” was the most common term, and 5 unique terms were observed among the 25 included in this group. Of the 148 terms in the diagnostic category, “diagnosis” was the most frequent (appearing 11 times), and 32 were unique. In the treatment category, “date treatment started” was the most common term, appearing 6 times; of the 72 terms in this category, 29 were unique. In the medical consultation category, “anthropometric data” was the term that appeared the most; of the 180 terms, 78 were unique.

Of the 17 terms, “disease malignancy” was the most common in the comorbidity category, with 8 unique terms. In hospitalization, of the 5 terms, “history of hospitalization” appeared most frequently (3 times). In the examination category, “general and specialized laboratory tests” were the most common among the 42 terms, of which 29 were unique. In the outcome category, “date of death” was the term that appeared the most, comprising 11 of the 57 terms, and in the others category, “the patient having previously provided a biological sample for research” was the most frequent term, comprising 2 of the 26 terms used in this category.

By combining these findings with the extraction, categorization, and synthesis techniques mentioned earlier, we used data science methods and clinical experience to design, structure, and recommend a fundamental global MDS for RD patient records in health care networks. It aims to comprehensively cover the data needed in clinical and management contexts. These summarized results can be found in Multimedia Appendix 3.

### Risk of Bias in Included Studies

The results of the risk of bias evaluations for the 20 included studies are presented in Multimedia Appendix 4. We reported the risk of bias evaluation using the STROBE statement standards and checklist [25]. The recommendations regarding the numerical indices can be found in tab 2 of Multimedia Appendix 4, STROBE statement recommendations. We used the “Unclear risk of bias” classification for cases where the recommendation did not apply to the study.

Using a matrix of 440 cells generated by crossing the 22 STROBE recommendations with the 20 selected studies, we found that 48.4% (213/440) of the items evaluated in all studies showed a low risk of bias and 39.1% (172/440) of these items had an unclear risk of bias. The remaining 12.5% (55/440) of the items assessed across all the studies had a high risk of bias.

In addition, the STROBE recommendations with the highest number of low risk of bias assessments for this set of studies were item 5, “Setting,” and item 14, “Descriptive data,” with 17 occurrences. The recommendation with the highest number of unclear risk of bias assessments was item 12, “Statistical methods,” with 14 occurrences. The STROBE recommendation that received the highest number of high-risk bias assessments was item 3, “Objectives,” with 12 occurrences.

## Discussion

### Principal Findings

We carried out this systematic review to elucidate the MDSs used for RD in health care network records in health care systems worldwide. The results showed a lack of terminological standardization of the concepts used in these MDSs. Different health systems may use different terms for the same concepts, hindering the interoperability, integration, and sharing of highly relevant information and knowledge to describe phenomena and generate health indicators.

The WHO recommends that health systems and networks use standardized terminology to exchange data, information, knowledge, and intelligence in health. Thus, health observatories that integrate population and epidemiological data require standard semantics to collect and organize this information to enable the generation of indicators capable of positively influencing public health policies [47]. Orphanet’s initiative also points to the need for a formal vocabulary to map and classify processes in RD. The lack of structured knowledge about RDs leads to problems, such as increased waiting time for a diagnosis and, consequently, difficulties in establishing an adequate treatment for these patients [48,49].

We encountered difficulties in the standardization and classification of the MDSs findings. Notably, the selected studies did not follow the same standard structure for classifying the data from their MDSs. Thus, we classified all data into 10 categories.

This process allowed us to summarize the information, verify its frequencies, perform statistical analysis, and analyze and identify synonyms (items with the same meaning, but described using different terms in specific MDSs). Clinical experts in RD and information and data scientists met in groups to verify and analyze these synonyms.

Other studies have proposed MDSs for specific groups of RDs [28-30,34-36,38-42,45,46], populations, and territories [2,29,33,43,44] based on different methodologies. For example, previous studies have proposed MDSs for RD in national and continental contexts. The European Commission’s Common Data Elements for Rare Disease Registration was developed for use by the RD registries across Europe. Similarly, the French national MDS was built nationally and defined through a national consensus for use in French RD centers [2]. However, to our knowledge, this is the first study to propose a data set for RD based on a systematic review that mapped MDSs used in different studies and could allow the application of the resulting data set to all RDs in unspecified populations.

Europe also has some examples of registries developed nationally and applied to an international consortium, such as the Fabry Disease Registry [50] and REGISTRY (for Huntington disease) [51]. However, neither provides open access to information regarding the data elements and structural evaluation. This limits researchers and practitioners who are not consortium members to access and adapt the registry to their context. In addition, these registries may limit the generalizability of the results to the entire affected population, because registered patients tend to have more access to medical care and treatment centers than the general population, especially in rural regions or countries with less developed health systems.

The Italian National Rare Diseases Registry [52] and Italian Neuromuscular Registry [53] are nationally developed registries intended to collect information on the prevalence and geographic distribution of rare and neuromuscular disorders, respectively. Both are considered effective models; however, their representativeness and generalizability of their data to other populations and countries may need to be improved.

A Spanish study addressed the importance of linking data to strengthen national RD registries [54]. However, the results were based on only 1 RD (amyotrophic lateral sclerosis, a motor neuron disease) and cannot be generalized to other diseases or datasets. In addition, this study highlights that coding errors and other inconsistencies may have affected the validity of the results. This is an important observation, because the accuracy of the results depends on the quality and consistency of the data. To address these limitations, researchers may adopt measures such as cross-checking data, manually reviewing selected cases, and statistical analysis. This evaluation was possible because the authors performed an association of records from different data sources, which occurred exclusively in this study.

Other studies have developed MDSs for RD designed for international applications; however, the MDSs were determined by a national committee. This is the case for the National Institutes of Health, National Center for Advancing Translational Sciences, and Global Rare Diseases Patient Registry Data common data elements [55]. All these studies present MDSs with limitations or restrictions because they cover only a specific RD, or their scope of application is restricted to a specific context or geographic region.

Therefore, the most innovative aspect of this study is the compilation of all the data used in MDSs for RD published worldwide. The fundamental MDS developed based on the analysis of this compilation by clinical and health data specialists can benefit services, assistance, research, and management in RD. Most national health systems and RD policies can also contribute to developing methods and processes and producing information. Thus, defining an MDS may improve data reliability to align strategies to enhance and manage health planning [11]. Although many policies describe comprehensive care in a network without a structured and standardized MDS, health centers cannot work in a coordinated manner.

Thus, the literature indicates the need to identify and implement common data elements [56] to improve care quality [57] and enable collaboration across different health care systems [58]. Our findings can help identify common standards and data elements to minimize the duplication of efforts and enhance the quality of patient records and data. This would lead to greater effectiveness of HISs and consequently, better patient outcomes. One of the recommendations for improving the quality of RD registries is to facilitate harmonization among the many institutions that collect patient information. Thus, it is essential to plan, design, and set up an MDS-based national information system and database for RDs that can provide and evaluate health indicators, promote network research, and foster public policies for RD care [11].

MDS is usually developed by reviewing the scientific literature, consulting with experts, and considering existing guidelines and recommendations. Although some of our findings report that they followed these processes in a combined or individual way, all were performed considering only a specific geographic context or addressed aspects related to a condition or subgroup of RD. Although a systematic review is an approach to synthesizing comprehensive scientific evidence, developing MDS involves reviewing the scientific literature, consulting with experts, and considering existing guidelines to identify essential data for RDs. Our systematic review approach provided a rigorous and transparent methodology to ensure the reliability and validity of the information collected and synthesized.

Identifying common trends and patterns can further simplify and streamline the information-gathering process, avoiding the need to develop and implement different data sets for each RD. This mapping saves time and resources, allowing for more efficient data collection. Sharing resources and knowledge plays a key role in supporting the research and development of therapies for rare conditions.

Variations in documentation practices in health systems can also lead to inconsistencies in data collection and standardization related to RD. These variations may include differences in the terminology used, coding systems, and data recording policies. Gaps in capturing these essential data elements can arise because of a lack of consensus on the most relevant information for understanding and studying RD. Existing data sets do not encompass all the aspects necessary for a comprehensive investigation, leading to the need for an MDS that identifies and incorporates essential data elements for scientific research in this field. This will allow for better comparison between different studies and registries.

Our study has some potential limitations. Among these, we highlight the methodological aspects of choosing the quality assessment method, publications, and generalization bias. In addition, we emphasize the potential for further investigations and possible outcome variations in different populations, contexts, and types of RD.

Owing to the originality and nature of the proposal, we did not define restrictions regarding the study design in our inclusion and exclusion criteria. Thus, all study designs were considered for inclusion in this systematic review. Therefore, selecting a method for assessing the quality of these studies is a nontrivial task, as such methods are usually designed to assess specific types of study designs. To mitigate this limitation, it was necessary to adopt the STROBE method in our proposal so that we could assess the quality of each of the selected studies using a standard tool. In addition, in the sequence, we cite possible biases and describe how to mitigate them.

This review may be affected by publication bias, as studies with positive results are more likely to be published than those with negative or inconclusive results. This can lead to a distorted view of the existing MDSs for RD. Therefore, we took several steps to reduce the risk of publication bias affecting our results, such as searching for multiple sources, recording the review, and contacting specialists. Recording the review process increases transparency, reduces the risk of publication bias, and promotes a more rigorous and impartial evaluation of available evidence. This helps avoid omitting relevant studies or data that may not align with the desired results. By keeping a comprehensive record, reviewers and readers can assess the potential impact of any publication bias and ensure that the review is conducted objectively and impartially [59].

Measures adopted to minimize the risk of excluding studies that were not published in scientific journals included the use of gray literature databases, prepublication repositories, clinical trial records, and scientific conferences. Finally, we contacted experts in the field to identify unpublished or ongoing studies that were not found in the systematic search.

Possible biases that may affect the generalizability and comparability of the findings must also be mentioned. Although there was no specific tool to mitigate the issue of generalization bias, it is worth highlighting that the studies included in this analysis were selected based on clear and well-defined criteria. During the data analysis process, we considered the differences between studies, such as population characteristics, study design, and data quality. Ultimately, our goal was to present the findings of our work clearly and transparently using valid instruments from the literature (Multimedia Appendix 5). We also aimed to highlight the limitations of our study and identify potential variations in the results based on robust evidence to provide valuable and relevant information for clinical practice and health decision-making.

### Conclusions

Our work discusses the difficulties in standardizing and classifying findings from MDSs for RD because of differences between studies. Clinical experts and data scientists have defined categories based on WHO guidelines to address this issue. This study aimed to compile MDS data from around the world and suggest a fundamental MDS for use in RD services, assistance, research, and management. The fundamental RD MDS designed in this study comprehensively covers the data needs in the clinical and management sectors.

The results can also help public policy makers achieve other aspects of their policies. For example, analyzing the state data produced by HISs is essential for qualifying and quantifying the care provided to people with RDs. It can also allow comparisons with local data regarding preventive actions provided to a community. This can improve decision-making at the managerial and local levels and contribute data that can inform strategic decisions at the national level [60].

In conclusion, the solid base of information compiled regarding MDSs for RD is a technical and social contribution to improving the health network’s ability to map its demands and better understand the public health scenario regarding rare conditions. Although the proposal of a fundamental MDS for RDs is highly relevant and, to our knowledge, unprecedented in the literature, we also suggest collecting data elements to be used in addition to this fundamental MDS, if necessary, for each group or type of RD, to increase the completeness and specificity of the data structure.

Owing to the high complexity of care processes involving RDs, structured information can significantly impact the quality of services offered to the population. A curated description of the methodology for developing an MDS for RD in low- and middle-income countries has not yet been published. We encourage further research in this context.

National data gathering for RD based on standard data sets to encourage interoperability by disseminating agreed-upon data-sharing guidelines can facilitate semantic data standardization. On an organizational level, it can assist institutions in establishing a registry, sharing deidentified data with research networks, and building specific and rich databases. On the basis of these results and the proposal of a fundamental MDS, we aimed to provide evidence-based subsidies to assist managerial and clinical RD processes in health systems.

## Appendix

Multimedia Appendix 1 General characteristics of the selected studies.

Multimedia Appendix 2 All variables collected.

Multimedia Appendix 3 Fundamental rare disease minimum data set.

Multimedia Appendix 4 STROBE (Strengthening the Reporting of Observational Studies in Epidemiology) evaluations.

Multimedia Appendix 5 PRISMA (Preferred Reporting Items for Systematic Reviews and Meta-Analyses) checklist.

## Abbreviations

HIS: health information system
LILACS: Literatura Latino-Americana e do Caribe em Ciências da Saúde
MDS: minimum data set
PCC: population, concept, and context
PRISMA: Preferred Reporting Items for Systematic Reviews and Meta-Analyses
RARAS: Brazilian National Network of Rare Diseases (Rede Nacional de Doenças Raras)
RD: rare disease
STROBE: Strengthening the Reporting of Observational Studies in Epidemiology
WHO: World Health Organization
